# Empathy and correct mental state inferences both promote prosociality

**DOI:** 10.1038/s41598-022-20855-8

**Published:** 2022-10-10

**Authors:** Konrad Lehmann, Anne Böckler, Olga Klimecki, Christian Müller-Liebmann, Philipp Kanske

**Affiliations:** 1 Clinical Psychology and Behavioral Neuroscience, Faculty of Psychology, Technische Universität Dresden, Chemnitzer Straße 46, 01187 Dresden, Germany; 2 University of Würzburg, Würzburg, Germany; 3 Max Planck Institute for Human Cognitive and Brain Sciences, Leipzig, Germany

**Keywords:** Psychology, Human behaviour

## Abstract

In a world with rapidly increasing population that competes for the earth’s limited resources, cooperation is crucial. While research showed that empathizing with another individual in need enhances prosociality, it remains unclear whether correctly inferring the other’s inner, mental states on a more cognitive level (i.e., mentalizing) elicits helping behavior as well. We applied a video-based laboratory task probing empathy and a performance measure of mentalizing in adult volunteers (*N* = 94) and assessed to which extent they were willing to help the narrators in the videos. We replicate findings that an empathy induction leads to more prosocial decisions. Crucially, we also found that correct mentalizing increases the willingness to help. This evidence helps clarify an inconsistent picture of the relation between mentalizing and prosociality.

## Introduction

In today’s world, we are faced with numerous challenges, from allocating the earth’s limited resources among a rapidly increasing population to managing a global pandemic, providing health care to people in need. Appropriately tackling these global, but also local, interpersonal issues requires cooperation, including acts of altruism and prosocial behavior. Prosocial behavior is commonly defined as any form of voluntary behavior that provides benefits to others, but is costly to the individual offering it^1^.

Potential motivational sources for altruistic acts can be various. Research has carved out a particularly strong association between empathy and the motivation to increase the wellbeing of another person, the so called empathy-altruism hypothesis^2–4^. Numerous studies have provided support for the link between empathy and altruism^5^. For example, a video-based empathy induction increased altruistic decisions in a Dictator Game, in which a participant can freely allocate a certain amount of money between herself and another person^6^.

However, empathy is often divided into an affective and a cognitive component that are well distinguishable both on a behavioral and neural level^7–9^. The affective component (a.k.a. affective empathy, or just empathy) is defined as the isomorphic or vicarious sharing of affective states^10,11^. In contrast, the cognitive component (a.k.a. cognitive empathy, mentalizing, Theory of Mind) is described as a rather deliberate understanding of others’ emotions, thoughts, desires, motives, etc.^12,13^. While the association between (affective) empathy and prosocial behavior is well-understood, the link between mentalizing, the cognitive component, and altruism is less clear.

It has been argued that a better understanding of others’ perspectives enhances one’s concern with their well-being and, thus, leads to altruistic acts^14^. Indeed, the developmental trajectories of increasingly complex mentalizing abilities parallel a trend towards a higher frequency of showing prosocial behavior^15,16^. Accordingly, children with better mentalizing skills as compared to their same aged peers allocate more resources to another person^17–20^. Likewise, in adults, higher mentalizing abilities are related to more altruistic decisions^21^. Neuroimaging studies showed that activity in the dorsomedial prefrontal cortex and the temporoparietal junction—regions commonly linked to mentalizing—can predict prosocial choices^22–24^. In contrast, there is also evidence for a nonexistent or even negative relation of mentalizing and prosocial behavior. For example, aggressive offenders that, by definition, showed severe antisocial behavior, do not exhibit altered mentalizing abilities^25^. Likewise, persons scoring high on the traits narcissism or psychopathy—that are both characterized by reduced prosocial behavior—appear to display normal^26,27^ or even slightly increased mentalizing performance^28^. Beyond that, it has also been shown and argued that the association between mentalizing and prosocial behavior underlies contextual variation (for reviews, see^29,30^). Prosocial behavior is diminished when the target of perspective-taking is too dissimilar to oneself, when the targets’ perspective bears the potential of negative self-evaluation, or when the context holds threats to the goals of the perspective taker^29,31,32^.

The findings regarding mentalizing and prosocial choices are, thus, not consistent and point to the notion of contextual nuance. Importantly, the reports are either based on theoretical considerations or on correlational studies, mostly relating interindividual differences in the capacity to mentalize to prosocial behavior. However, studies providing situation-specific, within-person links between these variables are lacking. To both, illuminate this gap, and gain further inside in contextual variability, we ask whether the accuracy of a mental state inference has a direct effect on the willingness to help. In other words, does correctly inferring the mental states of a specific person in a specific situation enhance prosocial decisions for this very person? Additionally, as the situation-specific relations between mentalizing and prosocial behavior have remained unexplored, the respective contributions of empathy-inducing situations and the accuracy of mental state inferences to prosociality are unknown. To address these questions, we applied a well-validated measure of mentalizing-performance and empathy (EmpaToM^33^) that has been utilized in a variety of studies (e.g.,^23,34–38^). In this task, narrators either tell an emotional or neutral, allegedly autobiographical story with or without ToM demands. Videos are followed by a valence rating (indicating empathic affect sharing) and by either a question regarding the narrators mental state (for videos with ToM demand) or a factual reasoning control question (for videos without ToM demand) (see Fig. 4). Subsequently, participants indicate their willingness to help the narrator. In line with the empathy-altruism hypothesis, we expected the willingness to engage in prosocial behavior towards the narrator following emotional videos (empathy induction) to be higher than after neutral videos and to increase with participants’ experienced negative affect. Crucially, we also expected correct mentalizing to go along with higher prosociality, that is, increased willingness to help after correctly inferred mental states as compared to incorrect mental state inferences. We expected the absence of such a difference for correctly versus incorrectly answered factual reasoning questions, serving as a tight control condition that allows to preclude potential influence of confounding factors such as attention or uncertainty.

## Results

### Empathy and prosocial decisions

**Figure 1:**
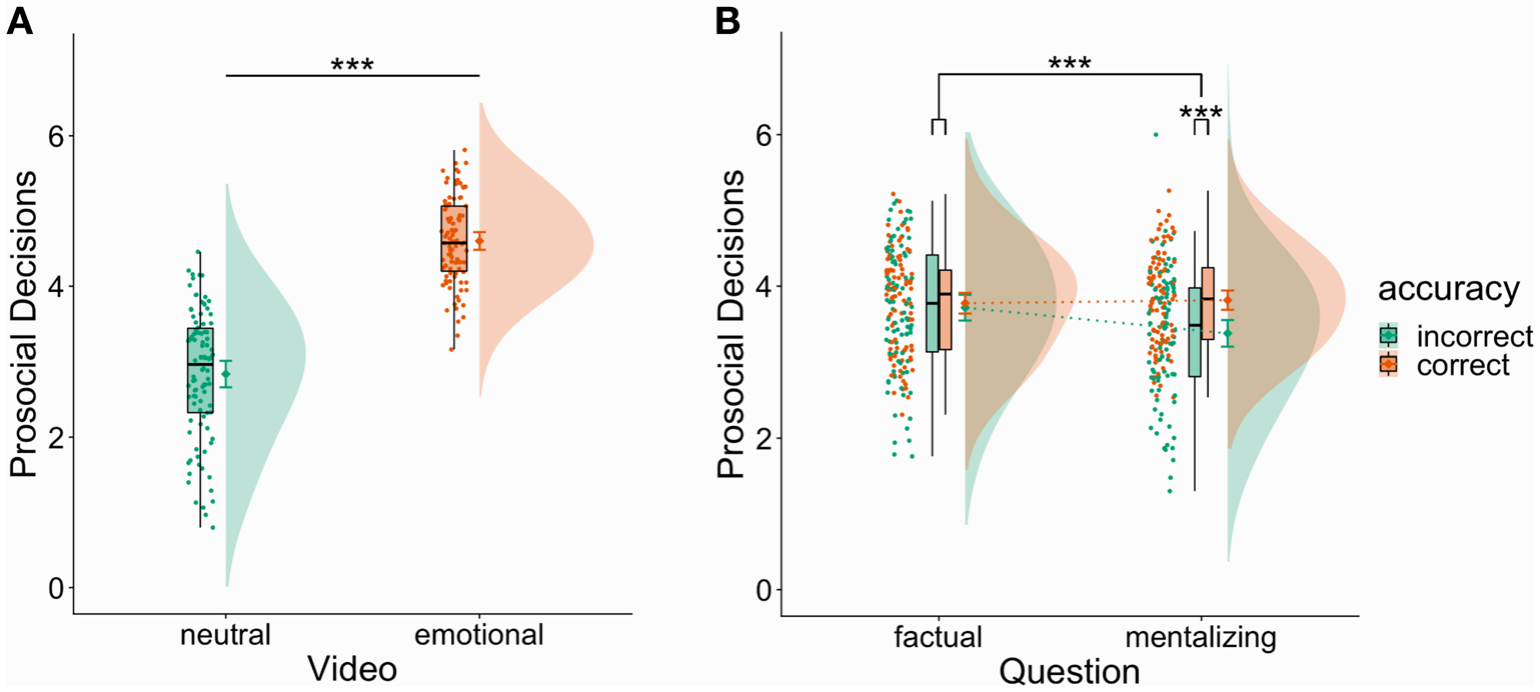
Results for prosocial decisions. Small dots represent individual data points, and dots with error bars show point estimates for the mean of the respective condition with 95% confidence intervals. Boxplots depict condition medians along with interquartile ranges, while shaded areas at the right of condition means show smoothed density distributions. (**A**) There was an increase in prosocial decisions following emotional vs neutral videos. (**B**) The interaction between accuracy (correct vs incorrect) and question type (factual vs mentalizing) on prosociality was significant (indicated by upper asterisk). Follow-up Bonferroni-corrected t-tests (lower asterisk) revealed an increase in prosocial decisions after correct vs incorrect mentalizing, whereas no difference in prosociality was observed after correct vs incorrect factual reasoning. *** indicates *p* < 0.001.

**Figure 2:**
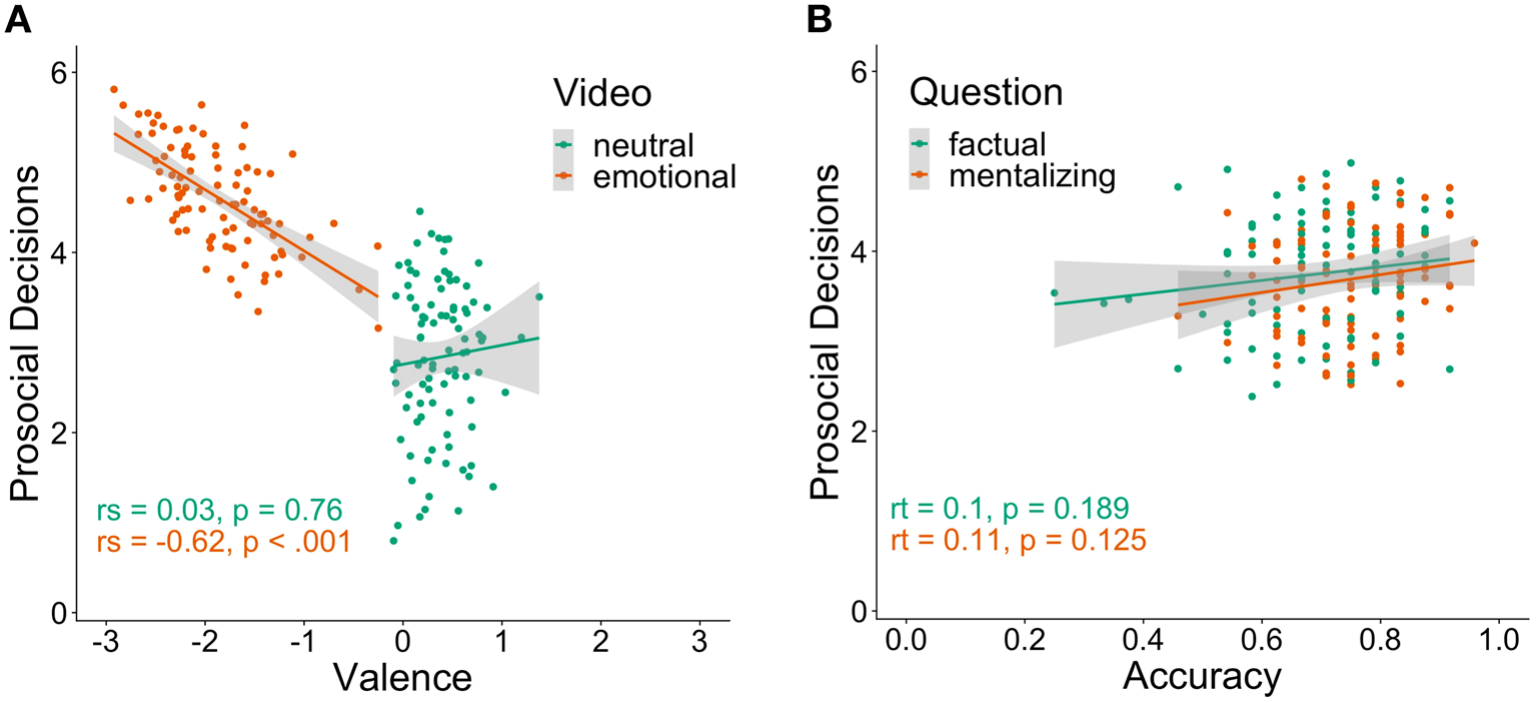
Scatter plots depicting interindividual differences. Grey-shaded areas represent 95% confidence intervals. (**A**) Relationship between valence ratings and prosocial decisions for the two video conditions (neutral vs. emotional), respectively. (**B**) Relationship between prosocial decisions and mean accuracy of the inference questions for the two question types (factual reasoning vs. mentalizing), respectively. rs: Spearman correlation coefficient, rt: Kendall’s tau correlation coefficient.

**Figure 3:**
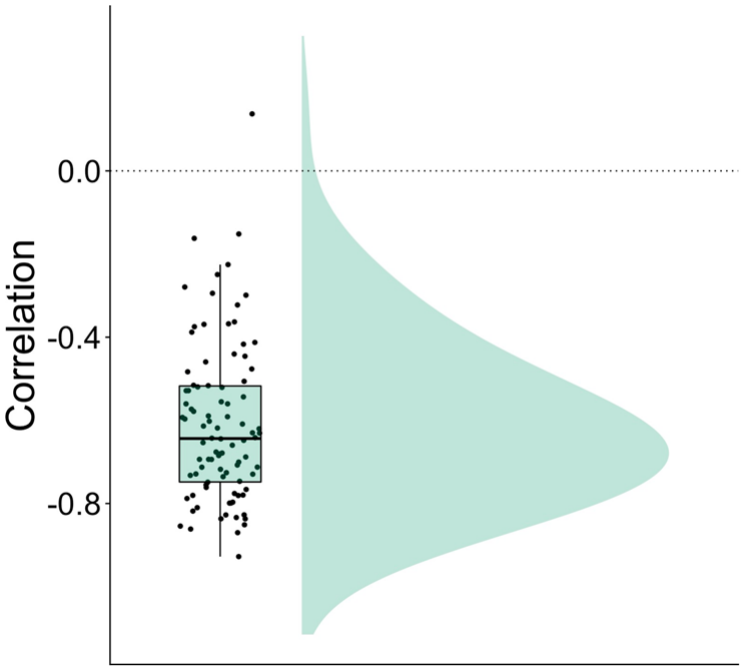
Result of the intraindividual correlations between valence and prosocial decision. Small dots represent individual data points. Boxplot depicts the correlation median along with interquartile ranges, while the shaded area at the right of the mean shows a smoothed density distribution.

Using a paired t-test, we assessed the effect of the empathy induction on prosocial decisions by comparing the means of the self-rated willingness to help the person in the video between the two video conditions (negatively emotional vs. neutral). In line with our hypothesis, we observed a large effect of the video condition (*M*_*µ1-µ2*_ = 1.76, 95% CI [1.59, 1.93], *t*(93) = 20.40, *p* < 0.001, Cohen’s *d* = 2.36, 95% CI [1.92, 2.81]), showing an increase in prosocial decisions following an emotional as compared to a neutral video (Fig. 1A). As a Shapiro–Wilk test showed a significant deviation of paired differences from normality (*W* = 0.97, *p* = 0.045), we additionally calculated a nonparametric Wilcoxon signed-rank test. This nonparametric test corroborated the results of the parametric paired t-test, showing that median ranks of prosocial decisions were higher for emotionally negative videos as compared to neutral videos (*V* = 17,760, *p* < 0.001). To investigate the relation between self-rated valence and prosocial decisions at an interindividual level, we calculated Spearman correlations between these two variables for the two video conditions (negatively emotional vs. neutral), respectively. We observed a strong negative correlation between valence ratings and prosocial decisions following emotional videos (*ρ* = -0.62, *p* < 0.001), indicating that stronger experience of shared negative affect was associated with increased helping behavior (Fig. 2A). We did not ascertain a relation between valence ratings and prosocial decisions following neutral videos (*ρ* = 0.03, *p* = 0.76). Using the R-package ‘cocor’^39^, we observed a significant difference between the correlations of valence ratings and prosocial decisions following emotional videos versus following neutral videos (Dunn and Clark’s *z* = −4.95, *p* < 0.001). Furthermore, we assessed intraindividual Spearman correlations between valence ratings and prosocial decisions for each participant and tested whether the correlations for the total sample differed from zero by using a Wilcoxon signed-rank test. We ascertained a high average intraindividual correlation between valence ratings and prosocial decisions (*Median =* −0.63, 95% CI [−0.59, −0.67], *V* = 1, *p* < 0.001) (Fig. 3). Results on the relation between compassion ratings and prosocial decisions are described in detail in Supplemental Materials S1.

### Mentalizing and prosocial decisions

Using a repeated measures ANOVA with two within-subjects factors (question type: mentalizing vs. factual reasoning; accuracy: correctly vs. incorrectly answered question), we tested the effect of correct (vs. incorrect) mental state inferences on prosocial decisions while controlling for factual reasoning inferences. In line with our hypothesis, we detected a two-way interaction between question type and accuracy of small effect size (*F*(1, 93) = 12.24, *p* < 0.001, *η*^2^_G_ = 0.02) (Fig. 1B). Bonferroni corrected pairwise t-tests indicated a difference in the willingness to help between correct (*M* = 3.82, 95% CI [3.69, 3.94]) and incorrect (*M* = 3.38, 95% CI [3.20, 3.55]) mental state inferences (*p* < 0.001) of medium effect size (Cohen’s *d* = 0.57, 95% CI [0.35, 0.79]), while there were no differences between correct (*M* = 3.77, 95% CI [3.64, 3.91]) and incorrect (*M* = 3.72, 95% CI [3.55, 3.89]) factual reasoning inferences (*p* = 1.00). To compare the effects of empathizing and mentalizing on prosocial decisions, we assessed the differences in effect sizes. The effect size of the comparison between prosocial decisions following emotional versus neutral videos (Cohen’s *d* = 2.36, 95% CI [1.92, 2.81]) was significantly bigger (*z* = 7.43 > 1.96 for two-tailed *z*-test) than the effect size of the comparison between prosocial decisions following incorrect versus correct mental state inferences (Cohen’s *d* = 0.57, 95% CI [0.35, 0.79]). As the ANOVA assumptions of normality of residuals (*W* = 0.99, *p* = 0.008) as well as homogeneity of variances (*F*(3, 373) = 3.72, *p* = 0.012) were violated, we computed a robust linear mixed-effects model (R-package robustlmm^40^) including random intercepts, fixed effects predictors (question type: mentalizing vs. factual reasoning; accuracy: correctly vs. incorrectly answered question) and their interaction. This robust linear mixed-effects model corroborated the results of our ANOVA by showing that the interaction of question type and accuracy significantly predicted prosocial decisions (*B* = 0.44, *SE B* = 0.10, *t* = 4.54, *p* < 0.001). To investigate the relation between the accuracy of the answered question and prosocial decisions at an interindividual level, we calculated Kendall correlations (due to ties in ranks) between the accuracy for the two question conditions (mentalizing vs. factual reasoning), respectively, and prosocial decisions. Here, we detected no significant correlations between prosocial decisions and the accuracy of mental state inferences (*r*_*τ*_ = 0.11, *p* = 0.125) and between prosocial decisions and the accuracy of factual reasoning inferences (*r*_*τ*_ = 0.10, *p* = 0.189) (Fig. 2B).

## Discussion

Employing a validated and naturalistic video-based assessment of empathy and mentalizing, we show that inducing empathy leads to a strong and reliable increase of prosocial decisions. These results provide support for the empathy-altruism hypothesis^2–4^. Put differently, when I am *feeling with* you I will probably help you. Moreover, and crucially, we demonstrate that correct mental state inferences support prosocial decisions towards the person whose mental states had been inferred. Put differently, when I am *thinking as* you, I will help you, too. Our results show that prosocial behavior is sensitive to the situational accuracy in the assessments of mental states. As such, they help to clarify the inconsistent picture of mentalizing effects on prosociality.

Assessing both social affect (empathy) and social cognition (mentalizing) in one task allows us to draw conclusions about their respective contributions to prosocial behavior. Here, we could ascertain that feeling empathy with a person is a more relevant factor to prosocial decisions than correctly inferring a person’s mental state. While the effect of the empathy induction (negatively emotional vs. neutral videos) on prosocial decisions was very large, the effect size of mentalizing accuracy (correct vs. incorrect mental state inferences) on prosociality was in a medium range. This finding resonates with the fact that affective states represent a more immediate activator of behavioral vigor than cognitions^41^.

The mechanistic explanations for engaging in prosocial behavior diverge between empathy and mentalizing. For empathy, it is not surprising that being confronted with negative feelings of another person (and sharing this feeling) led to prosocial choices that would, in consequence, also reduce one’s own negative feeling. Interestingly, positive social affect, the feeling of compassion, led to an increase of prosocial decisions as well (see Supplementary Material). In contrast to empathic emotion sharing or even empathic distress, compassion has been defined as the feeling of warmth and care for others^11^ and represents a rather other-directed component, while empathic distress is more related to a reduction of one’s own negative feeling (cf.^42^). In our data, compassion exhibited a stronger correlation with prosocial decision than empathy, which fits strong motivational components ascribed to compassion^11^.

For mentalizing, on the other hand, it has been argued that a better understanding of others’ perspectives enhances one’s concern with their well-being^14,20^. Another possible cause might be that it is more likely to infer someone’s mental state correctly when being more similar to the other, as one’s prior expectations about their behavior are met more frequently. Fulfilled expectations are less energy consuming and more rewarding, while the feelings of reward, again, promotes prosocial behaviors^43^. In this vein, similarity in traits between two persons also predicts the quality of a friendship^44^, which itself is characterized by reciprocal prosocial acts^45^. This explanation resonates well with the concept of social discounting, stating that the amount of money a person is willing to give another person is varying as a function of social closeness between them^46^. Another possible explanation for an engagement in prosocial behavior following accurate mental state inferences might point towards effort justification^47^. It could be argued that more effort in the process of mentalizing leads to more accurate mental state inferences and hence to an increase in prosocial behavior. However, we can refute this argument as the same effect would also apply to the accuracy of factual reasoning questions, which did not differ between correctly and incorrectly answered questions.

Beyond our main results, we also showed on a level of interindividual differences that the more affective responses were shared (as indexed by a lower rating of emotional valence) the more participants were inclined to make prosocial decisions. This applied to the context of negative emotional videos, while we did not observe such a correlation in neutral videos. Our results are in line with recent evidence reporting a correlation between the extent of empathy and the amount of money given to another person in a dictator game^6^. Interestingly, we could extend this notion by showing this relation on an intraindividual level. In the present study, persons showed more pronounced prosocial decisions when they stated that they felt more negative emotions after being faced with the suffering of others. In contrast to ratings of empathy, on an interindividual level, there was no correlation between mentalizing performance and prosocial decisions. As such, this speaks against an often-reported correlational evidence between mentalizing performance and prosocial behavior^17–21^and rather supports the notion of a non-existent association. Here, the results of our within-subjects design could deliver evidence that the contextual dependence in prosocial decisions between correct versus incorrect mentalizing might mask a more strong relationship on an interindividual level. However, on the other hand, in our task the accuracy of mentalizing is accumulated over a series of binary outcomes (correct versus incorrect) and, thus, captures less variability as compared to the emotional valence rating. Hence, the probability for observing a relationship between mentalizing and prosocial decisions in our task is diminished, which might explain the discrepancy between our results and other findings.

Beside the strength of our study, we acknowledge that our results are based on an observational stance of our participants, being not engaged in reciprocal and dynamical interaction. It has been shown that behavior diverges between observational and interactive settings and that comparisons between those two settings have to be made with caution^48,49^. In an interactive setting, mentalizing evolves from a one-way street–where the mental states of a target person are inferred by a study participant–towards nested recursive mentalizing between interactive partners in the sense of ‘I think that you think that I think…”^50^. This recursivity might reduce the motivation to act prosocially as, for example, the interactive partner could harbor negative intentions towards the self, which can lead to defensive and ‘antisocial’ instead of prosocial acts. However, our findings can inform future studies that examine truly interactive contexts. Another objection might be that the way we assessed prosocial behavior does not capture altruistic behavior in the narrow sense because prosocial decisions in our task were hypothetical and, thus, did not benefit others at the own cost. However, such a hypothetical assessment of prosocial behavior has been shown to strongly load on a factor of prosocial behavior that is altruistically motivated, and that includes also charitable donations and given money in a dictator game^51^. Another limitation of our study might be seen in the composition of our sample, which fits to the description western, educated, industrialized, rich, and democratic^52^. Conclusions on other populations should be drawn with caution and we encourage the investigation of our question in populations that are different from the current.

In conclusion, our results provide further support for the empathy-altruism hypothesis as empathy inducing videos increased the extent of prosocial decisions. Importantly, an increase in affect sharing both intra- and interindividually predicted readiness for helping. Moreover, and crucially, the current study shows that prosocial decisions are sensitive to situational accuracy of mental state inferences. This adds an important insight to an inconsistent and mainly correlational picture of the link between mentalizing and prosociality. In this way, the present study illuminates how distinct processes of empathizing and mentalizing shape prosocial decisions. These results might be a building block in understanding prosocial behavior and, therefore, may contribute to foster altruism, tackling both personal and global challenges.

## Methods

### Participants

The final sample consisted of 94 volunteers (64 female, 30 male; mean age = 24.78, *SD* = 6.72; age range = 18–65) who were recruited via local advertisements via flyer or e-mail. As this data acquisition occurred at the start of a brief meditation training study, for reasons of safety, participants who stated they had epilepsy, respiratory insufficiency, post-traumatic stress disorder or a diagnosis of schizophrenia were excluded. The initial sample size amounted to 100 participants and was determined for the meditation training via calculations for observing moderate correlational effect sizes with a power of 80% and a significance level of 5%. For the present study, the sample size is suited to detect small effects in two-way (2 × 2) repeated measures design^53^. Due to technical issues with the paradigm, data for the first six participants were lost, yielding our final sample of 94 participants. For their participation in the overall study, participants were compensated with €30 or course credit. Ethics approval was granted by the ethics committee of Technische Universität Dresden (reference number: EK 180052018) and the study was carried out in accordance with the declaration of Helsinki. We obtained written informed consent from all participants prior to participation.

### Stimuli and procedure

**Figure 4:**
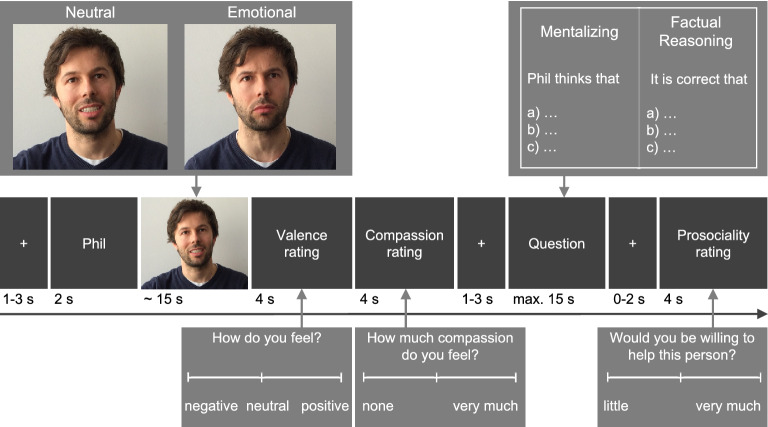
EmpaToM trial sequence. In each trial, the participant is presented with an autobiographical narration that could vary on two dimensions (emotionality of the video: neutral vs. emotionally negative; ToM requirement: videos with and without ToM demand). After each video, participants rated their own affect (valence rating) and their compassion for the person in the video. This is followed by, a question either requiring mental state inference (for videos with ToM demand) or factual reasoning (for videos without ToM demand), and a prosociality rating (adapted from Kanske et al.^33^). Note that the exemplary images depicted in this figure are not based on the original video stimuli used in the EmpaToM task but, due to license restrictions, have been replaced with re-staged images showing (a meanwhile much younger version of) one of the authors.

We used the EmpaToM task—a well-validated paradigm to assess mentalizing, empathy, and compassion^33^. In this task (see Fig. 4), participants were presented with a short video-clip (~ 15 s.) in which a female or male narrator either told a neutral (e.g., selling items on eBay) or emotionally negative (e.g., one’s sister suffering from bowel cancer), allegedly autobiographical story that either does or does not impose ToM demand. After viewing the video, participants indicated their current feeling state on a continuous visual analogue scale (from negative to positive, which we coded as −3 to 3 with 720 possible increments), to assess empathic affect sharing (i.e., a negative valence rating after an emotionally negative video). Subsequently, using continuous visual analogue scales ranging from none to very much participants rated the extent to which they felt compassion with the narrator in the video. Next, to test inferences about the video content, a single choice question with three response options was displayed to which participants had a maximum of 15 s time to answer (correct answers were scored with 1, while incorrect answers were scored with 0). Importantly, in videos with ToM demand, the question targeted mental state inferences of the narrators (e.g., “Phil thinks that…”); in videos without ToM demand, the question concerned factual reasoning (e.g., “It is correct that…”) about non-mental content of the story. After having answered this question, we assessed prosocial decisions towards the narrator by having participants indicate if they were willing to help the person in the video (on a visual analogue scale from little to very much, which we coded as 0 to 6 with 720 possible increments). This kind of hypothetical allocation of resources is commonly used in behavioral economics and also resembles the approach of the social value orientation scale^54^ or the social discounting task^46^, which have been shown to represent altruistically motivated prosocial behavior^51^. Except for the additional prosociality rating, the task was carried out in the same way as validated in the original large-scale study^33^. The paradigm encompassed a total of 48 videos of 12 narrators (male and female) and 12 videos per each of four conditions (video: neutral vs. emotional; question: mentalizing vs. factual). Exemplary video stories and questions can be found in Supplement S2.

### Data analysis

Data analysis was carried out using MATLAB (MathWorks, Inc., Natick, Massachusetts, United States) and R^55^. Graphs depicting density plots were prepared using raincloud plots^56^. Data and analysis code for this study can be found on the Open Science Framework (https://osf.io/tu2gj/?view_only=f285308c035d451ca2fce5f3788f97e3).

### Empathy and prosocial decisions

In order to replicate previous findings on prosociality following an empathy induction, we conducted a paired t-test of prosocial decisions between the neutral and emotional video condition that have been shown to differ strongly in ratings of empathic affect sharing^34^. As the paired differences were not normally distributed, we additionally calculated a nonparametric Wilcoxon signed-rank test. To investigate effects of interindividual differences, we conducted Spearman correlations between participant-wise mean valence- and prosociality ratings for the two video conditions (negatively emotional vs. neutral), respectively. Moreover, to test for a correlation of valence- and prosociality ratings on an intraindividual level, we calculated a Spearman correlation between the valence- and prosociality ratings for every participant and conducted a Wilcoxon signed-rank test against zero.

### Mentalizing and prosocial decisions

In order to investigate our hypothesis that correct mental state inferences support an increase in prosociality, we conducted a repeated measures ANOVA of the prosocial decisions with the two within-subjects factors accuracy (correctly vs. incorrectly answered question) and question type (mentalizing vs. factual reasoning). We expected a two-way interaction in such a way that prosocial decisions are increased after correct mental state inferences as compared to incorrect inferences while such a difference is absent in factual reasoning. As ANOVA assumptions were violated (normality of residuals and homogeneity of variances), we conducted a robust linear mixed-effects model to corroborate our ANOVA findings. The mixed-effects model included random intercepts, fixed effects predictors factors (question type: mentalizing vs. factual reasoning; accuracy: correctly vs. incorrectly answered question) and their interaction. To examine interindividual differences, we applied a Kendall correlation between person-wise mean accuracy- and prosociality ratings for the two question conditions (mentalizing vs. factual reasoning), respectively. We used a Kendall correlation because, compared to Spearman correlations, it can handle ties in ranks.

## Supplementary Information

Supplementary Information.

## Data Availability

We report how we determined our sample size, all data exclusions, all manipulations, and all measures in the study, and we follow the journal article reporting standard^57^. The datasets generated and/or analysed during the current study are available in the Open Science framework, https://osf.io/tu2gj/?view_only=f285308c035d451ca2fce5f3788f97e3. Stimulus material can be obtained upon request from the corresponding author. This study’s design and its analysis were not pre-registered.
