# Supplementary material for: Empathy and correct mental state inferences both promote prosociality
Source: Sci Rep. 2022 Oct 10;12:16979. doi: 10.1038/s41598-022-20855-8 (PMC9550828; doi:10.1038/s41598-022-20855-8)
Supplement: Supplementary file 1 — Supplementary Information. [file 41598_2022_20855_MOESM1_ESM.docx]

**Supplementary Material**

**Supplement S1**:Compassion and prosocial decisions

To investigate the relation between interindividual differences regarding prosocial decisions and self-rated compassion, we calculated Spearman correlations between these two variables for the two video conditions (negatively emotional vs. neutral), respectively. We observed strong correlations between compassion ratings and prosocial decisions following emotional videos (*ρ*  = .84, *p* < .001) and following a neutral videos (*ρ* = .67, *p* < .001). Using the R-package ‘cocor’ (Diedenhofen & Musch, 2015), we observed a significant difference between the correlations compassion rating and prosocial decisions following emotional videos versus following neutral videos (Dunn and Clark’s *z* = 2.80, *p* = 0.005). Comparing the respective relations between compassion, valence and prosocial decisions, we ascertained significant differences between *valence rating and prosocial decisions* and *compassion rating and prosocial decisions* both following emotional (Dunn and Clark’s *z* = -10.37, *p* < 0.001) and neutral videos (Dunn and Clark’s *z* = -5.91, *p* < 0.001). This indicates an independence of the two measures valence and compassion.

**Figure S1**

*Scatter plot depicting the relationship between compassion and prosocial decisions*


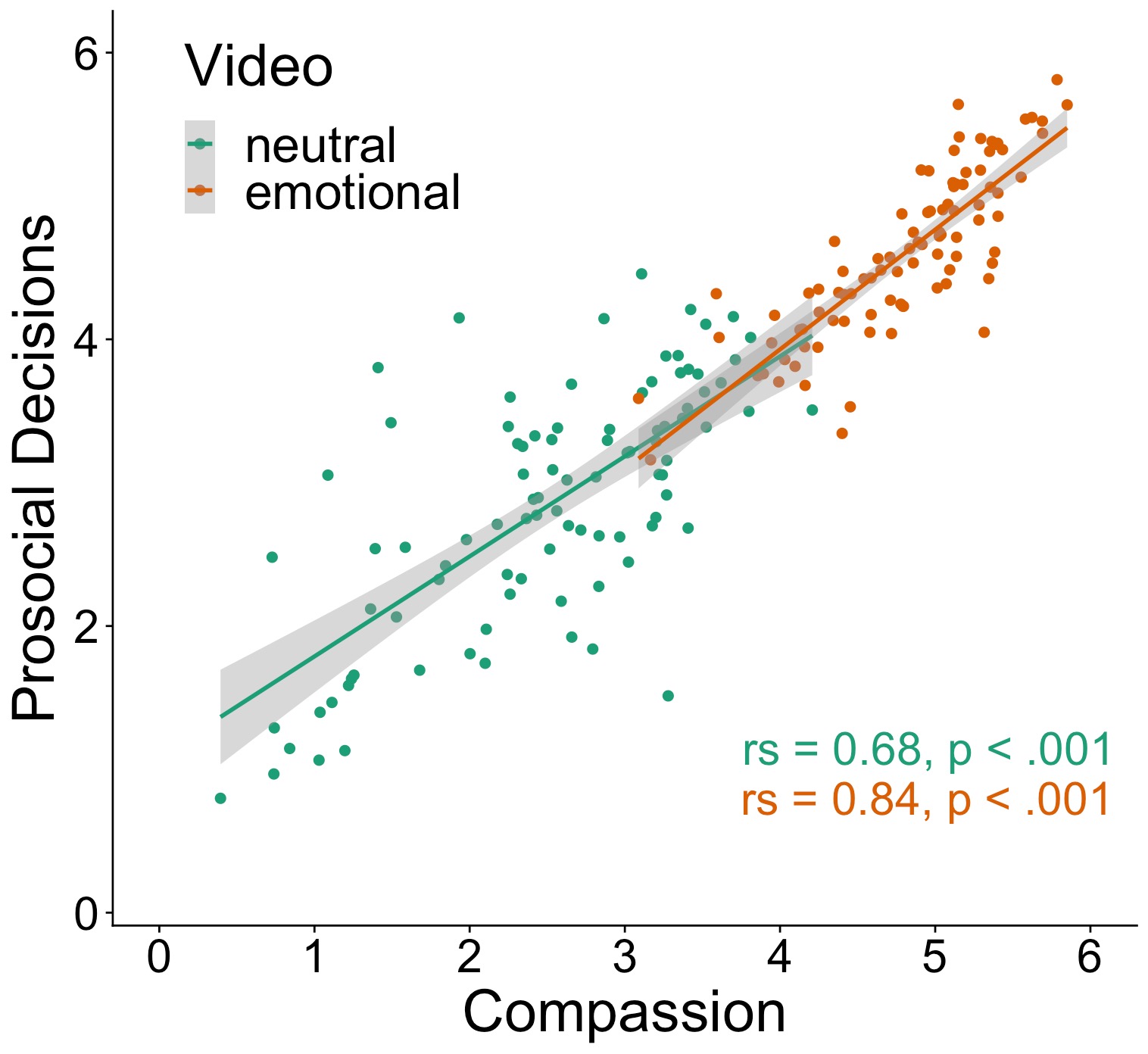


*Note.* Grey-shaded areas represent 95% confidence intervals. rs: Spearman correlation coefficient.

**Supplement S2**: Example stories and questions for each experimental condition

Example 1: Anna

*Neutral nonToM*

„Hm… well, … that evening, I cooked. I prepared one of those 3-course meals. And my boyfriend invited his sister. She brought a nice red wine from her vacation in France. And then there was my former flatmate. Well, I think we were sitting in the kitchen until one, one thirty.“

It is true

- that Anna’s boyfriend was at the party. **(correct)**
- that Anna has been to France before and has brought some red wine.
- that Anna has been living with friends for quite a while.

*Neutral ToM*

„My best friend Laura recently went to the movies with my brother. And she loves these cartoon movies, and my brother also thought the movie was great. He wants to watch another one of those with her right next week. That doesn’t really sound like him… he used to be more into the action stuff.“

Anna thinks

- that her brother fell in love with her best friend and this is why he watches cartoon movies with her. **(correct)**
- that her brother’s being in love entirely changed his taste in movies.
- that her brother plans to also watch action movies with her best friend.

*Emotional nonToM*

„We’ve been together for five years now, and it wasn’t like we didn’t like each other any more. But… at some point we just couldn’t stop fighting. And once, he got so mad at me, he… hit me in the face. I just couldn’t really go on after that.“

It is true

- that Anna met her ex-boyfriend at least five years ago. **(correct)**
- that Anna and her boyfriend don’t see each other often since they started dating.
- that Anna’s ex-boyfriend was often violent.

*Emotional ToM*

„My sister was diagnosed with bowel cancer a year ago and the odds aren’t great. But you have to cling to something, don’t you. Her doctor recently suggested a new treatment to her, but she refuses to try. It just makes me wanna cry.“

Anna thinks

- that her sister gave up hope and doesn’t want treatment anymore. **(correct)**
- that her sister wants to look for an appropriate treatment herself.
- that her sister would probably be saved by the new treatment.

Example 2: Hannes

*Neutral nonToM*

„I am doing quite a lot of reading at the moment, mainly German classics, Thomas Mann, for example. I like it and I also need it for a class. And when I am through, I want to dive into Russian literature.“

It is true

- that Hannes has read more German than Russian literature in the past. **(correct)**
- that Hannes needs to read a lot for his thesis.
- that Hannes studies philosophy, which is why he needs to read all the classics.

*Neutral ToM*

„It was a gift, Katharina gave me these ridiculously expensive musical tickets. I sold them, but told her that I really enjoyed the show. When I did, somehow her smile froze … “

Hannes thinks

- that Katharina wanted to go to the musical together with him. **(correct)**
- that Katharina does not believe he liked the musical so much..
- that he would have really liked the musical.

*Emotional nonToM*

„I got beaten up in the subway pretty bad, by three guys. They circled me and kicked me… And nobody helped.“

It is true

- that Hannes could not run from the offenders. **(correct)**
- that Hannes had bumped into the men in the subway and then they beat him up.
- that Hannes screamed for help, when the men beat him up.

*Emotional ToM*

„My father is an alcoholic. Often times he doesn’t come home for days and then the police brings him. My mom is worried like crazy. And I am also worried, but I cannot come home each time ...“

Hannes thinks

- that his mother expects him to take better care of her. **(correct)**
- that his father knows how much his mother is worried.
- that his father will always find his way back home eventually.
